# Characteristics of the postural stability of the lower limb in different visual states of undergraduate students with moderate myopia

**DOI:** 10.3389/fphys.2022.1092710

**Published:** 2023-01-04

**Authors:** Zhaoxin Huang, Xiaofei Xiao

**Affiliations:** School of Rehabilitation Medicine, Binzhou Medical University, Yantai, Shandong, China

**Keywords:** postural stability, moderate myopia, y-balance test, attention, EMG

## Abstract

**Objective:** To explore the characteristics of lower limb postural stability in undergraduates with moderate myopia in three different visual states.

**Methods:** Twenty male undergraduate students were recruited to complete respectively the static and dynamic postural stability tests under eyes-closed, myopia (taking off their glasses immediately) and corrected vision conditions. A three-dimensional force platform (Bertec, United States) was used to test static postural stability, which calculated the total path length of the Center of Pressure (COP), path length in the antero-posterior (A/P) and medio-lateral (M/L) directions, COP area, SampleEntropy (SampEn), and low-, medium-, and high-frequency spectrum energies. Dynamic postural stability was tested using the Y-balance test, and the Y-balance test scores were calculated. The Vicon three-dimensional motion capture system (Oxford, United Kingdom) measured the maximum flexion angles of the ankle, knee, and hip joints. The electromyography (EMG) root mean square (RMS) and integral EMG (iEMG) of the tibialis anterior and lateral gastrocnemius of the lower extremity were simultaneously measured using wireless surface electromyography (Noraxon, United States).

**Results:** The SampEn-A/P and SampEn-M/L of corrected vision state higher than myopia and eyes-closed states, and myopia state larger than eyes-closed state (χ^2^ = 51.631, *p* < .001). The original and standard scores of the anterior, postero-medial and comprehensive values of the three visual states had significant differences (F = 32.125, *p* < .001). The original and standard values of postero-lateral corrected vision and myopia were larger than those of eyes-closed states (F = 37.972, *p* < .001). The maximum flexion angles of the ankle and knee joints were in the following order: corrected vision, myopia and eyes-closed (F = 10.93, *p* < .001). The iEMG and RMS had significant differences in the three different states (χ^2^ = 12.700, *p* < .001) in the all directions of YBT.

**Conclusion:** Compared with corrected vision, the stability of static posture in the state of myopia was decreased, and the postural regularity was more regular. The dynamic postural stability in the state of myopia was also lower than that corrected vision, and the activation and work of ankle muscles were also increased.

## 1 Introduction

Postural stability refers to the ability to keep the body’s center of pressure stable within the supporting area (Elboim-Gabyzon et al., 2022). The sensory inputs that are required to maintain postural stability in humans include vision, vestibular, and proprioception. When the visual information is blocked, the static postural stability swing of the human body will increase by 20%–70% (Isotalo et al., 2004). According to a recent prediction, the global population of people with myopia is expected to reach 5 billion by 2050 (Holden et al., 2016). Since the outbreak of the COVID-19 pandemic, the incidence and severity of myopia among undergraduate students appear to be on the increase (Fang and Guo., 2021). If myopia is not controlled promptly, it could easily progress to high myopia, which leads to a series of pathological changes, such as retinal detachment, glaucoma, cataract, and even permanent visual impairment or blindness (Lord and Dayhew., 2001). When myopia progresses to a certain extent, it caused blurring of vision, decreased contrast sensitivity and stereoscopic sense, and decreased postural stability (Ikuno, 2017). However, there are currently few studies on the effects of myopia on postural stability, and existing studies have focused on the ability to maintain static balance. According to Willis et al. (2013) found that people with myopia had more static postural instability than those with normal vision. Bae et al. (2020) found that the static postural stability of people with uncorrected vision (without glasses) was significantly lower than that of those with corrected vision. Myopia was also significantly higher than corrected visual acuity in the middle- and high-frequency spectral energy of the somatic system. Sayah et al. (2016) found that people with myopia were more unstable than those with emmetropia in terms of visually induced postural responses. The postural stability includes both static and dynamic aspects, and testing static stability alone cannot fully explain the characteristics of postural stability in people with myopia. The risk of falls in humans is influenced to a greater extent by dynamic postural stability than by static postural stability, and most falls occur during dynamic motion (Ray et al., 2008; Jeter et al., 2015). Due to the lack of accurate visual input and feedback after visual impairment, accidental injuries including falls, are more likely to occur during dynamic motion. Most studies have focused on epidemiology and its influencing factors (Liu et al., 2022; Pan, 2022).

When vision is impaired, the sensory input of visually impaired individuals appears as the vestibular and proprioception compensative mechanism (Schwesig et al., 2011). Labanca et al. (2021) compared with eyes-opened, the ankle muscle group co-activation and ankle strategy of young people were more obvious when the posture was unstable, the neuromuscular system could improve the stability by regulating the activity of the ankle muscle group in the eyes-closed state. However, it is unclear whether myopia affects muscle activation.

Therefore, the objective of this study was to explore the characteristics of static and dynamic postural stability in undergraduate students with moderate myopia in eyes-closed, myopia, and corrected vision states. In this research would assume: 1) The stability of static posture in the myopia state was lower than that in the corrected vision state, which was better than that in the eyes-closed state. 2) The stability of dynamic posture in the myopia state was lower than that in the corrected vision state, which was better than that in the eyes-closed state. 3) In the dynamic stability test, the activation intensities of the tibialis anterior (TA) and gastrocnemius (GA) were in the following order: eyes-closed, myopia, and corrected vision states.

## 2 Materials and methods

### 2.1 Participants

From April to June 2022, 20 male undergraduate students with moderate myopia (age: 19.5 ± 1.10 years; height: 1.75 ± .06 m; weight: 72.64 ± 13.63 kg; visual acuity: 4.33 ± −80 D (based on a verbal declaration and used the International Standard Logarithmic Visual Acuity Chart (Chen et al., 2020); and lower limb length: .90 ± .56 m) in Binzhou Medical University were recruited.

The inclusion criteria were as follows: 1) the age was >16 years; 2) the corrected vision was normal, and the better visual acuity was moderate myopia (-3.0D to -6.0D) (Goes and Delbeke., 2022); 3) non-sports major students; and 4) participants who signed informed consent forms.

The exclusion criteria were as follows: 1) abnormal musculoskeletal function, or proprioception, vestibular function, disturbance of consciousness, severe cardiovascular disease, *etc.*; 2) history of neuropathy or administration of drugs that may affect balance; 3) damage to the inner ear and another sensory system that may affect balance; and 4) difference in astigmatism, strabismus, and binocular vision >100°; 5) those who have received balance training or regular physical activity (regular physical activity was defined as undergoing vigorous-intensity physical activity for >20 min or moderate-intensity physical activity for >30 min, at least once weekly (Park et al., 2022) in the past 6 months.

The study followed the tenets of the Declaration of Helsinki and was approved by the ethical review board of the scientific research project of the Medical Ethics Committee of Binzhou Medical University (Ethics approval No: 2021–233).

### 2.2 Procedure

#### 2.2.1 Static postural stability test

The static postural stability was tested using a three-dimensional force platform (Bertec, United States) with a sample rate of 1000 Hz to collect static postural stability data (Gómez-Landero et al., 2021). Before the test, the participants performed warm-up exercises. The participants stood barefoot in the center of the force platform, while looking forward with the distance between the feet being equal to their shoulder width, and their upper limbs hung naturally on both sides of the body, and they remained motionless for 25 s. The data were taken from the middle 15 s, and the first 5 s and last 5 s were removed (Pryhoda et al., 2020). Measurements were taken three times under eyes-closed, myopia (tested immediately after taking off the glasses), and corrected vision states, and testing in this order. The average values were calculated. After each measurement, the participants rested for at least 1 min.

The indicators for evaluating the static postural stability include the linear indicators (the total path length of the Center of Pressure (COP), et al.), the non-linear indicators (SampleEntropy (SampEn)), and frequency spectral energy (low-, middle-, and high-frequency spectral energy). SampEn is the statistical regularity of the time series, with low (high) values indicating more (less) regularity (Stins et al., 2009). It is generally believed that the lower the SampEn, the more regular the COP signal regularity. The lower the automation of postural control, the higher the requirement for attention (Roerdink et al., 2011; Kędziorek and Błażkiewicz., 2020). Postural stability is maintained through the integration of sensory information provided by the visual, proprioceptive, and vestibular systems (Loughlin and Redfern., 2001). Those systems have different time delays in their control pathways, enabling their relative afferent contributions to be studied by identifying characteristic COP frequency responses (Daniels et al., 2019). The frequency spectral energy can be obtained by performing a fast Fourier transform, which enables the researcher to convert wave graphs of COP movement as represented by amplitude (power) and time into a relationship between amplitude (power) and frequency (spectrum); the power included for each frequency is defined as the frequency spectral energy (Tanaka et al., 2020). It is generally agreed that visual information of postural sway at low frequency (0–.3 Hz), vestibular information of postural sway at intermediate frequency (.3–1 Hz), and proprioceptive information of postural sway at high frequency (1–3 Hz) (Kanekar et al., 2014).

#### 2.2.2 Dynamic postural stability test

**FIGURE 1:**
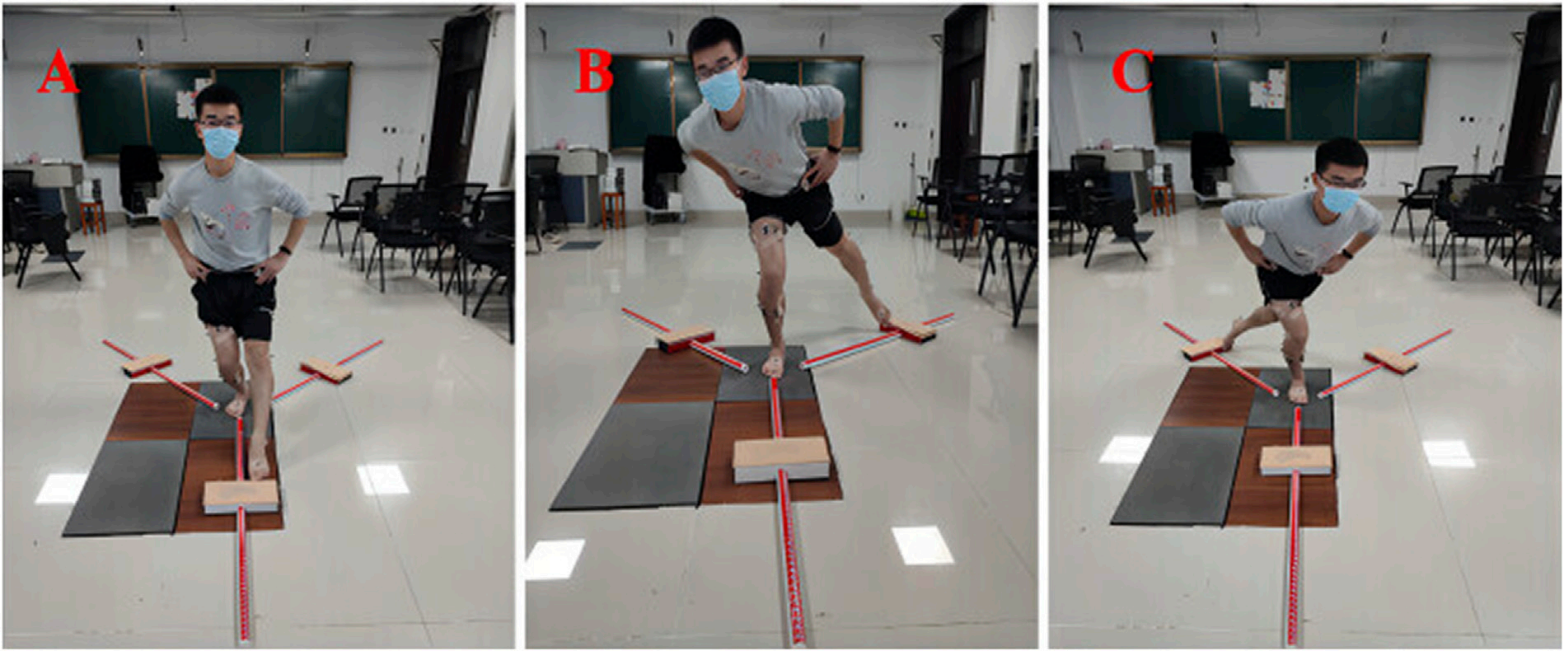
The picture of the subject during the YBT. **(A)** The anterior direction of the YBT. **(B)** The postero-medial direction of the YBT. **(C)** The postero-lateral direction of the YBT.

The dynamic postural stability test used the Y-balance test (YBT) exercise kit to collect dynamic postural stability data (Salas-Gómez et al., 2022). The YBT includes the anterior A) (Figure 1A), postero-medial (PM) (Figure 1B), and postero-lateral (PL) (Figure 1C) measurements. During the YBT, the VICON MX motion analysis system (Oxford, United Kingdom), with a sample rate of 100 Hz (Tang et al., 2022) was used to record the maximum flexion angles of the ankle, knee, and hip joints. Surface electromyography (Noraxon, United States) with a sample rate of 1500 Hz (Yu et al., 2022) was used to test the EMG signal characteristics of the TA and lateral GM. Recording the EMG signal from the beginning of the extension of the leg to the extension to the farthest.

The disposable surface electrodes for EMG was attached to the participants after the warm-up exercises. First, the body hair was removed, the skin was disinfected with 75% alcohol, after which the skin was wiped repeatedly to reduce impedance. The EMG was then attached to the belly of the muscles along the direction of the muscle fibers after the skin was dry, and the sensor was connected and fixed. The Maximum Voluntary Contraction (MVC) was tested using the manual resistance methods (Balasukumaran et al., 2020). The muscle group around the ankle of the dominant leg was tested twice for 5 s each time at intervals of 10 s. The average value of the Root Mean Square (RMS) in the two MVC tests of each muscle was used to standardize the RMS (Mueller et al., 2018). The landmarks of the Vicon motion capture system were modeled according to the Plug-in-Gait of the lower body, and 16 marker balls were affixed to the subjects’ landmarks.

First, the dominant leg (the leg the participants first choose to kick a ball (van Melick et al., 2017) was determined. While the participants stood on the dominant leg, the non-dominant leg was used as the forward extension leg. The participants stood barefoot in the center of the YBT exercise suite, and the dominant toe was aligned directly in front of the central horizontal line, while the participants held their waist with both hands. The right leg was tested counterclockwise, while the left leg was tested clockwise. The next direction was changed after three measurements in each direction. All measurements had an accuracy of ±.5 cm (Zafar et al., 2020). Before the formal test, the participants were asked to make six attempts in each direction of each leg to familiarize themselves with the test movement. To avoid the influence of learning, the participants were required to take a 5-min break before taking the formal test. If the participants failed to stretch out in six attempts, the direction was marked as 0 cm.

Measurements were taken three times under the eyes-closed, myopia (tested immediately after taking off the glasses), and corrected vision states, and testing in this order. The average values were calculated. After each measurement, the participants rested for 1 min.

Note: The following failures were not included in the data analysis: loss of balance while standing on one leg, obvious movement of the standing foot, landing support of the extended foot, and failure to return to the original position.

#### 2.2.3 Evaluation index

The linear indicators were: the total path length of the COP, path length in the antero-posterior (A/P) and medio-lateral (M/L) directions, and the COP area were measured. The non-linear indicators were: SampEn, low-, middle-, and high-frequency spectral energy. The original and standard values of the extension distances in the three directions of the YBT (the comprehensive value of the extension distance; the maximum angles of the ankle, knee, and hip joint movement; RMS; and integral EMG value (iEMG)) were also evaluated.

### 2.3 Data process and analysis

The EMG signals were smoothly filtered using Noraxon MR3 software, and were rectified along with band-pass filtering (10–500 Hz) (Coratella et al., 2021). Finally, COP and EMG signal data were calculated and processed using MatLab 2020 software (The Math Works, Natick, MA, United States).

### 2.4 Statistical analysis

*A priori* power analysis was performed with G*Power 3.1.9.7 software. Using the Repeated Measures Analysis, setting the alpha error at .05, the Power at .80, and comparing three states in one group with an effect size (f = .50), showed that a minimum of 15 participants. Finally, we recruited 20 participants. The statistical calculations were carried out using SPSS 22.0 (IBM, Armonk, NY, United States) software for statistical analysis of the data. The normality of each data set was evaluated by the Shapiro-Wilk normality test. Quantitative data that were normally distributed were expressed as mean ± standard deviation, and F-tests were used to determine differences in the three states, i.e., the scores of YBT and angles of joints. This experiment was a compatibility group experiment to explore the differences between the same sample under three different visual states. It was a randomized block design and also belongs to the related sample design. Repeated measurement analysis of variance was not in line with normality, expressed by Median values (25 percent of quartiles, 75 percent of quartiles), using non-parametric Friedman’s test. Chi-squared tests were used to determine differences in the three states, i.e., the index of COP, RMS, and iEMG. A Bonferroni correction factor was applied if significant. The criterion of statistical signification was set at .05.

## 3 Results

The linear indexes showed corrected vision lower than eyes-closed state (*p* < .001) (Table 1). There were no differences between eyes-closed and myopia state or between myopia and corrected vision state (*p* > .05) (Table 1). The sample entropy of non-linear indicators showed that visual factors had a significant effect on postural regularity (*p* < .001) (Table 1). The middle- and high-frequency spectrum energies during eyes-closed and myopia states were higher than those of corrected vision states (*p* < .001) (Table 1).

**TABLE 1:** Comparison of static postural stability of lower limbs in different visual states (Median values (25 percent of quartiles, 75 percent of quartiles)).

Project	Eyes-closed	Myopia	Corrected vision	*χ* ^*2*^-value	*p*-value	Post-hoc comparisons		
						Eyes-closed VS. myopia	Eyes-closed VS. corrected vision	Myopia VS. corrected vision
						*p*-value	*p*-value	*p*-value
Total path length of COP(m)	0.24 (0.20, 0.29)	0.21 (0.15, 0.27)	0.21 (0.16, 0.24)	12.100	0.002	—	0.002	—
Path length of COP_A/P_(m)	0.18 (0.13, 0.23)	0.16 (0.12, 0.21)	0.15 (0.12, 0.19)	5.853	0.007	—	0.020	—
Path length of COP_M/L_(m)	0.11 (0.09, 0.14)	0.10 (0.07, 0.14)	0.09 (0.07, 0.13)	11.100	0.004	—	0.003	—
COP area (cm^2^)	616.25 (286.96, 1360.10)	577.50 (175.12, 1278.00)	488.87 (192.26, 890.41)	7.900	0.019	—	0.022	—
SampEn-A/P	0.85 (0.58, 0.99)	1.02 (0.85, 1.26)	1.38 (1.17, 1.61)	51.631	<0.001	<0.001	<0.001	<0.001
SampEn-M/L	0.76 (0.55, 0.94)	1.02 (0.82, 1.22)	1.24 (.93, 1.43)	76.960	<0.001	<0.001	<0.001	<0.001
Low spectrum energy (%)	67.56 (54.95, 74.38)	72.81 (63.25, 81.13)	81.51 (69.41, 84.30)	23.070	<0.001	0.003	<0.001	0.004
Middle spectrum energy (%)	17.60 (14.81, 24.67)	18.61 (12.06, 20.53)	11.90 (9.84, 17.85)	14.678	<0.001	—	<0.001	0.002
High spectrum energy (%)	14.30 (10.50, 24.07)	10.53 (6.96, 18.23)	6.40 (5.46, 11.74)	21.700	<0.001	—	<0.001	0.008

Note: —, *p* > 0.05.

The YBT showed that there were significant differences in the original and standard scores of A, PM, and comprehensive values among the three visual states (*p* < .001) (Table 2). The scores from highest to lowest was as follows: corrected vision, myopia, and eyes-closed (*p* < .001) (Table 2). The original and standard scores of PL between myopia and corrected vision state had no differences (*p* > .05) (Table 2).

**TABLE 2:** Comparison of extension scores of the Y-balance test in different visual states (mean ± standard deviations).

Project		Eyes-closed	Myopia	Corrected vision	*F*-value	*p*-value	Post-hoc comparisons		
							Eyes-closed VS. myopia	Eyes-closed VS. corrected vision	Myopia VS. corrected vision
							*p*-value	*p*-value	*p*-value
Standard value (%)	A	55.72 ± 9.30	62.77 ± 8.04	67.81 ± 8.24	56.656	<0.001	<0.001	<0.001	<0.001
	PM	92.13 ± 16.09	101.51 ± 11.73	108.86 ± 13.01	38.751	<0.001	<0.001	<0.001	0.003
	PL	86.19 ± 17.30	101.50 ± 15.14	104.37 ± 13.30	39.368	<0.001	<0.001	<0.001	—
	Composite	78.01 ± 12.90	88.59 ± 10.51	93.68 ± 10.53	78.429	<0.001	<0.001	<0.001	<0.001
Original Value (cm)	A	50.73 ± 7.42	57.31 ± 7.03	61.48 ± 7.10	50.938	<0.001	<0.001	<0.001	<0.001
	PM	84.13 ± 12.15	91.75 ± 10.29	98.07 ± 9.53	32.125	<0.001	0.003	<0.001	0.003
	PL	78.70 ± 15.32	91.56 ± 11.96	94.33 ± 9.41	37.972	<0.001	<0.001	<0.001	—
	Composite	213.57 ± 31.21	240.61 ± 25.93	253.89 ± 22.74	71.325	<0.001	<0.001	<0.001	<0.001

Note: —, *p* > 0.05.

The knee, and ankle joints under three visual states had prominent differences in the A direction (*p* < .001) (Table 3). The maximum flexion angle from largest to smallest was as follows: corrected vision, myopia, and eyes-closed. The hip joint had no difference A direction (*p* > .05) (Table 3). The hip and knee joints in three visual states were significant differences in the PM direction (*p* < .001) (Table 3). The joints angles had no differences between myopia and corrected vision state in the PL (*p* > .05) (Table 3).

**TABLE 3:** The Y-balance test comparison of maximum flexion angle of lower limb joint in three visual states (mean ± standard deviations).

Project		Eyes-closed	Myopia	Corrected vision	*F*-value	*p*-value	Post-hoc comparisons		
							Eyes-closed VS. myopia	Eyes-closed VS. corrected vision	Myopia VS. corrected vision
							*p*-value	*p*-value	*p*-value
A	ankle	26.95 ± 8.91	31.56 ± 10.30	35.23 ± 9.39	17.408	<0.001	0.028	<0.001	0.013
	knee	45.24 ± 18.90	56.36 ± 21.89	64.85 ± 22.00	28.545	<0.001	0.001	<0.001	0.001
	hip	26.14 ± 16.00	25.61 ± 17.47	27.76 ± 21.69	0.409	0.584	—	—	—
PM	ankle	22.41 ± 11.82	24.48 ± 11.05	26.42 ± 10.94	10.933	<0.001	0.021	0.002	—
	knee	43.56 ± 20.05	52.11 ± 18.34	63.56 ± 20.42	34.913	<0.001	0.002	<0.001	<0.001
	hip	58.13 ± 19.34	69.95 ± 17.04	79.38 ± 18.54	32.627	<0.001	<0.001	<0.001	0.002
PL	ankle	20.25 ± 9.79	25.27 ± 12.18	28.01 ± 14.60	27.900	<0.001	0.003	<0.001	—
	knee	28.19 ± 16.88	40.50 ± 19.30	42.04 ± 21.68	15.268	<0.001	<0.001	<0.001	—
	hip	49.22 ± 15.08	65.44 ± 19.28	67.70 ± 18.55	24.490	<0.001	<0.001	<0.001	—

Note: —, *p* > 0.05.

The iEMG and RMS had significant differences in the three different states (*p* < .01) (Tables 4, 5) in the all directions of YBT.

**TABLE 4:** The Y-balance test RMS comparison of lower limb muscles in three visual states (Median values (25 percent of quartiles, 75 percent of quartiles)).

Project		Eyes-closed	Myopia	Corrected vision	*χ* ^*2*^-value	*p*-value	Post-hoc comparisons		
							Eyes-closed VS. myopia	Eyes-closed VS. corrected vision	Myopia VS. corrected vision
							*p*-value	*p*-value	*p*-value
A	TA	1.11 (0.65, 2.07)	0.78 (0.41, 0.53)	0.70 (0.37, 1.23)	30.900	<0.001	<0.001	<0.001	—
	GM	3.11 (1.48, 4.80)	2.60 (1.13, 3.70)	2.58 (1.08, 3.37)	16.300	<0.001	0.022	<0.001	—
PM	TA	0.83 (0.75, 2.24)	0.78 (0.50, 1.45)	0.60 (0.43, 1.31)	22.900	<.001	0.002	<0.001	—
	GM	2.08 (0.92, 3.53)	1.85 (0.78, 3.01)	1.55 (0.59, 2.35)	30.700	<0.001	0.008	<0.001	0.034
PL	TA	0.94 (0.72, 1.98)	0.72 (0.50, 1.69)	0.68 (0.40, 1.55)	30.700	<0.001	0.008	<0.001	0.034
	GM	2.16 (0.94, 3.19)	1.32 (0.65, 2.50)	1.08 (0.55, 2.10)	36.400	<0.001	0.002	<0.001	0.034

Note: —, *p* > 0.05.

**TABLE 5:** The Y-balance test iEMG comparison of lower limb muscles in three visual states (Median values (25 percent of quartiles, 75 percent of quartiles)).

Project		Eyes-closed	Myopia	Corrected vision	*χ* ^*2*^-value	*p*-value	Post-hoc comparisons		
							Eyes-closed VS. myopia	Eyes-closed VS. corrected vision	Myopia VS. corrected vision
							*p*-value	*p*-value	*p*-value
A	TA	58.09 (38.48, 99.73)	42.02 (27.62, 84.69)	37.99 (21.66, 71.36)	30.700	<0.001	0.008	<0.001	0.034
	GM	28.41 (19.38, 46.05)	25.26 (16.05, 42.81)	17.65 (11.82, 41.51)	19.900	<0.001	0.022	<0.001	—
PM	TA	54.13 (35.83, 71.08)	44.82 (27.78, 55.60)	39.21 (25.54, 50.69)	22.800	<0.001	0.013	<0.001	—
	GM	26.00 (17.10, 43.64)	19.36 (15.39, 33.23)	15.87 (11.66, 35.39)	28.900	<0.001	0.022	<0.001	0.022
PL	TA	65.90 (38.89, 104.33)	48.12 (33.74, 85.89)	37.64 (30.66, 64.98)	25.900	<0.001	0.008	<0.001	—
	GM	21.83 (14.69, 43.33)	18.61 (11.51, 40.38)	15.32 (10.74, 41.21)	12.700	0.002	0.005	0.008	—

Note: —, *p* > 0.05.

## 4 Discussion

The purpose of this research was to explore the effects of different visual states on postural stability. The results showed that when maintaining static postural stability, the regularity of postural control in the myopia state was more regular than corrected vision. Under different visual acuity, the dynamic postural stability from stable to unstable was corrected vision, myopia, and eyes-closed. The activation and the work of the TA and GA from largest to smallest was as follows: eyes-closed, myopia, and corrected vision. Compared with corrected vision, the dynamic postural stability of myopia was decreased and the activation of ankle muscles was increased.

Analysis of the linear index revealed that the static postural stability in the state of corrected vision was better than that of eyes-closed. Entropy analysis of the non-linear index sample showed that when maintaining static postural stability, the postural control regularity under different visual conditions was as follows: eyes-closed, myopia, and corrected vision. That was, the attention required in the eyes-closed state for maintaining postural stability was higher than the states of myopia and corrected vision. Although the linear analysis method can describe and analyze the development and changes in postural control (Yamagata et al., 2017), the non-linear time series analysis method can provide a richer supplementary description of the potential developmental changes in postural control. SampEn is one of the various types of entropy measures that is used to evaluate the variation in postural swing with time, behavior regularity, and predictability. It is generally believed that the lower the SampEn, the more regular the COP signal regularity. The lower the automation of postural control, the higher the requirement for attention (Roerdink et al., 2011; Kędziorek and Błażkiewicz., 2020). Therefore, when visual information is impaired, automation of body postural control and adjustment is reduced. To maintain postural stability, myopia required more attention than corrected vision. In a study of postural control in children and adolescents of different ages, Kiefer et al. (2021) found that there was no difference in the length of the COP trajectory in the young group with eyes open, but the regularity of postural control in the eyes-closed state was higher than that in eyes-opened states. However, studies on the effects of vision on static balance had revealed that the static balance of college students in normal vision states was better than that of those eyes-closed (Bednarczuk et al., 2021). In this study, COP data were only different in corrected vision and eyes-closed states, but there was no difference between COP data of myopia and those of other states. This may be because the participants recruited in this study were young male undergraduates, and the task of standing on two feet under different visual states was relatively simple and not challenging for them (Sarto et al., 2022). In a study on the effect of corrected vision on postural stability, Bae et al. (2020) found that the static postural stability of uncorrected visual acuity (without glasses) was significantly lower than that of the corrected state. People with myopia were more unstable than those with emmetropia in terms of visual-evoked postural response (Sayah et al., 2016). When myopia progresses to a certain extent, it causes blurring of visual information and decreases contrast sensitivity and stereoscopic perception (Ikuno, 2017), which leads to a decline in postural stability. Myopia and eyes-closed states also led to a significant increase in the middle and high-frequency spectral energy. Bae et al. (2020) found that myopia increased the middle- and high-frequency spectral energy of somatosensory compared with corrected vision, which also proved that myopia had a negative effect on static postural stability.

In this study, we observed that the original and standard values of the dynamic postural stability of the lower extremity were significantly different among the three visual states. The dynamic stability in the corrected vision state was higher than the myopia and eyes-closed states, and myopia state higher than eyes-closed. However, the stability of PL myopia was similar to that of corrected vision, which was higher than that of the eyes-closed state. In the YBT, the lower limb joint movement strategies were different according to different directions and vision. Muscle activation was also different, and the TA and GA were more obviously affected by vision in the three directions. The activation and work of the TA and GA from largest to smallest was as follows: eyes-closed, myopia, and corrected vision states. Vision is the main sensory modality required by the body to maintain postural stability (Grace et al., 2012). When the body was in the states of eyes-closed or myopia, the immediate disappearance or inaccuracy of visual information altered sensory input and decreased postural stability. Vision had a greater influence on dynamic stability in the A and PM directions of the YBT, in which postural stability in myopia was worse than that in the corrected vision state but better than that in the eyes-closed state. It was further explained that blurred or impaired vision would have a negative effect on postural stability. However, in the PL direction, the postural stability in the states of myopia and corrected vision were similar but higher than that of the eyes-closed states. This may be because the PL structure of the YBT was not similar to the A direction, where the stretching baffle could be seen directly. The head and neck were generally in a neutral position during PL stretching, regardless of whether the vision was corrected, and there was a deviation between the afferent and feedback of PL spatial positioning and movement accuracy.

In the YBT, vision had a great influence on the range of motion of the ankle, knee, and hip joints of the lower extremity, in which there were significant differences in the maximum flexion angles of the ankle and knee joints under the three types of visual acuity, and the angles in the corrected vision state were larger than the myopia and eyes-closed states, and the myopia state larger than eyes-closed. Differences were observed between the PM and PL components in the hip joints. In adults, the extension scores of the YBT were moderately positively correlated with the ankle dorsiflexion angle (Kang et al., 2015a; Kang et al., 2015b), and the hip joint was mainly reflected in the PM and PL components (Kang et al., 2015a; Kang et al., 2015b), which also showed a consistent trend in this study.

The visual system also plays an important role in postural control. Schmidt et al. (2021) compared the effects of eye-opening, eyes-closed, wearing sunglasses, and a dark environment on postural stability, and found that dynamic stability was worse when the eyes were closed, and the activation of the TA and GA was more obvious. Gao et al. (2020) also found that after visual deprivation, the work of TA and GM was higher than those before visual deprivation. Although the test methods were different in the YBT, especially in the A and PM directions, it was also found that the TA and GA were more obviously affected by visual acuity. The activation and work of the TA and GA from the largest to the smallest was as follows: eyes-closed, myopia, and corrected vision states. The neuromuscular control system includes four aspects: muscle strength, reaction time, proprioception, and postural control. When postural stability was disturbed, the neuromuscular control system enhanced postural control ability by regulating muscle activation and coordination (Pan and Jin., 2012). Combined with the range of motion of the ankle joint in the three visual states, it was found that when visual information was limited, the neuromuscular control system increased the activation and work of the TA and GA, and improved proprioception of the muscles around the ankle to meet the need for dynamic stability. In a study of the relationship between adolescent patients with myopia and abnormal body posture, Wang and Feng (2022) found that abnormal body posture increased the risk of myopia. Frequent reminders by teachers and increased physical activity could reduce cases of myopia caused by abnormal body posture. In this study, compared with corrected vision, myopia caused a decrease in dynamic postural stability and increased the activation of lower limb muscles, while the risk of falls was more related to dynamic stability (Ray et al., 2008; Jeter et al., 2015). Myopia may increase the risk of falls; therefore, it is particularly important to correct body posture, enhance physical exercise, and promptly prevent and control the degree of myopia.

This study mainly discussed the characteristics of postural stability in moderate myopic undergraduates in the state of myopia immediately after taking off their glasses. In the future, research should focus on a comparative analysis of the postural stability of myopia immediately after taking off the glasses and after adaptation (after a long period of adaptation). It should also explore the muscles around the knee and hip.

## 5 Conclusion

Moderate myopia has a negative effect on human postural stability. Compared with the corrected vision state, the stability of static posture in the state of myopia was decreased, and the postural regularity was more regular. Dynamic postural stability in the myopia state was also worse than that in the corrected vision state, and also increased activation and the work of muscles around the ankles.

## Data Availability

The original contributions presented in the study are included in the article/supplementary material, further inquiries can be directed to the corresponding author.
